# Transitional Care from Hospital to Cardiac Rehabilitation During COVID-19: The Perspectives of Older Adults and Their Healthcare Providers

**DOI:** 10.1177/23743735231213757

**Published:** 2023-11-13

**Authors:** Cecilia Flores-Sandoval, Joseph B. Orange, Bridget L. Ryan, Tracey L. Adams, Neville Suskin, Robert McKelvie, Jacobi Elliott, Shannon L. Sibbald

**Affiliations:** 1 Faculty of Health Sciences, 6221 Western University, London, Canada; 2 Faculty of Health Sciences, School of Communication Sciences and Disorders and Canadian Centre for Activity and Aging, 6221 Western University, London, Canada; 3 Departments of Family Medicine and Epidemiology and Biostatistics, Centre for Studies in Family Medicine, London, Canada; 4 Department of Sociology, 6221 Western University, London, Canada; 5 Department of Family Medicine, Schulich School of Medicine & Dentistry, 6221 Western University, London, Canada; 6 St. Joseph's Hospital Cardiac Rehabilitation & Secondary Prevention Program, London, Canada; 7 St. Joseph's Health Care, London, Canada; 8 Lawson Health Research Institute, London, Canada; 9 School of Health Studies, 6221 Western University, London, Canada; 10 Interfaculty Program in Public Health, 6221 Western University, London, Canada

**Keywords:** older adults, transitional care, cardiac rehabilitation, patient engagement, COVID-19

## Abstract

Transitional care to cardiac rehabilitation during the pandemic was a complex process for older adults, with additional challenges for decision-making and participation. This study aimed to explore the perspectives of older adults and health providers on transitional care from the hospital to cardiac rehabilitation, focusing on patient participation in decision-making. A qualitative exploratory design was used. Semi-structured interviews were conducted with 15 older adults and 6 healthcare providers. Document analysis and reflexive journaling were used to support triangulation of findings. Six themes emerged from the data, related to insufficient follow-up from providers, the importance of patients’ emotional and psychological health and the support provided by family members, the need for information tailored to patients’ needs and spaces for participation in decision-making, as well as challenges during COVID-19, including delayed medical procedures, rushed discharge and isolating hospital stays. The findings of this study indicated a number of potential gaps in the provision of transitional care services as reported by older adults who had a cardiovascular event, often during the first few weeks post hospital discharge.

## Introduction

Cardiovascular disease is one of the most common causes of chronic disease and the second leading cause of death in Canada.^1^ Cardiac rehabilitation is a comprehensive program designed to help individuals with cardiovascular disease regain their health through patient education, lifestyle changes, medical assessment and exercise training.^2^ Older adults with cardiovascular disease benefit greatly from cardiac rehabilitation^3^; however, transitions from one health care setting to another can result in patient vulnerability, often aggravated by lack of coordination among healthcare providers and unclear discharge instructions.^4^

Transitional care is a set of actions that ensure the coordination and continuity of healthcare as individuals transfer between different locations or levels of care.^5^ Care transitions can be very challenging because they encompass the integration and coordination of care across different health care sectors and providers that are involved in the patient's circle of care.^6^

Given that the complexity of transitional care may increase for older adults as they navigate the healthcare system,^7^ and since their participation in decision-making during transitional care is sometimes missing,^8,9^ the present study aimed to explore the perspectives of older adults and their healthcare providers on transitional care from hospital to cardiac rehabilitation during the COVID-19 pandemic, with a special focus on the patient decision-making.

## Method

### Design

This study used a qualitative exploratory design grounded in the constructivist paradigm that considers human action as situated culturally and socially, with multiple constructed realities, versus adopting a single reality.^10^

### Participant Selection and Recruitment

Purposive sampling was used to select participants^11^ from a cardiac rehabilitation unit in [name of place]. Older adults (≥65 year) were eligible to participate in the study if they had been hospitalized within the past year for a myocardial infarction, had undergone percutaneous coronary artery revascularization, or coronary bypass graft surgery. Older adults were recruited via phone call, while providers were recruited via a mass email prepared by the research personnel of the cardiac rehabilitation program. Providers interested in the study contacted the primary investigator to participate. A web-based survey tool (Qualtrics) was used to give participants access to the letter of information, and to record written consent. Hardcopy letters of information were also available via postal mail, with the option of recorded verbal consent.

### Ethics Approval

This research study was approved by Western University (116533) and Lawson's Health Research Institute (R-20-557) Ethics boards.

### Data Collection and Analysis

In-depth semi-structured interviews (30-60 min) were conducted by the first author via phone call or Zoom, audio recorded and transcribed. Older adult participants also were asked to complete a brief demographic form using Qualtrics. See Table 1 for sample interview questions.

**Table 1.** Sample Interview Questions.

**Questions for older adults:**
Did you feel ready to leave the hospital when you were told that you could go home? *Probe:* If not, why? If yes, why? What would an ‘ideal’ transition in care from hospital to cardiac rehabilitation, look like for you? Were you encouraged and supported to talk about your concerns, personal preferences and/or experiences during your first appointment at the cardiac rehabilitation program? *Probe:* If yes, in what ways were you encouraged and supported? Tell me about a decision that was made during your first appointment (eg, prescriptions, new routines). *Probes:* How was that decision made? How do you feel about this process? Who typically makes decisions for your care? *Probes:* Is this how you prefer decisions to be made? Does your provider ever make decisions on your behalf? *Probe:* If yes, give me an example. How does that make you feel? What role/roles do your caregivers or family members have during your appointments? Would you prefer to talk to your provider alone? *Probe*: If yes, why? If not, why not?
**Questions for healthcare providers:**
How are first appointments after hospital discharge typically booked? Can you tell me about the communication with the hospital? Explain how you enact ‘patient participation’ in your practice in terms of decision-making. How does this ‘patient participation’ change, depending on the age of the patient? What happens when older adults cannot participate or are not willing to participate in decision making? What are some of the challenges in promoting participation in health care decision-making by older adults? What can you tell me about the impact of age-related factors, such as poor vision or hearing problems, on decision-making? If you had no constraints or limitations, what would an ‘ideal’ first appointment look like post-hospitalization for an older adult?

To enhance rigour and to triangulate findings, document analysis, reflexive journaling and member checking were used.^12^ Documents included patient education guides, such as lifestyle workbooks and exercise diaries. Reflexive notes, with no patient identifiable data, were taken by the primary investigator.

Data analysis was conducted using ongoing deductive and inductive analysis. The processes of data collection and analysis were simultaneous. The primary investigator (CFS) transcribed interviews verbatim line-by-line and analyzed participant transcripts using NVivo12 software. Due to COVID-19-related public health measures and restrictions in Ontario and the medical vulnerability of participants, all data were collected remotely. Methods such as field immersion and participant observation on-site were not possible. Member checking sessions were conducted with six participants who had agreed to be re-contacted at the end of the study. Birt and colleagues^13^ model was adapted and used to conduct member checking sessions.

## Results

### Participants

A total of 21 semi-structured interviews were conducted, 15 interviews were conducted with older adults and 6 with providers. Older adults were 10 male and 5 female participants (*M *= 72.73 year, *R *= 65-82 year). Hospitalizations occurred between August 2020 and January 2021, with an average hospital stay of 4.6 days. The majority of participants were married and living with their spouse at the time of the interview. Please see Table 2 for demographic information.

**Table 2.** Demographic Information of Older Adult Participants.

Gender	
Male (n = 10)	67%
Female (n = 5)	33%
**Age**	
65–70 (n = 5)	33%
71–76 (n = 6)	40%
77–82 (n = 4)	27%
**Level of Education**	
Secondary (n = 3)	20%
Post-secondary (n = 12)	80%
**Length of Hospital Stay**	
1–5 days (n = 9)	60%
6–14 days (n = 4)	27%
Preferred not to share (n = 2)	13%

Six interviews were conducted with 4 female and 2 male healthcare providers (*M *= 39 year, *R *= 30-62 year). Providers were physicians, nutritionists, nurses, psychologists and kinesiologists, with at least five years of experience working with older adults. Given the small number of healthcare providers who work in cardiology and cardiac rehabilitation, additional demographic information was not requested to protect their privacy and prevent identification of participants.

### Findings

Data analysis generated six themes as presented in Table 3. To preserve confidentiality, non-identifiable random numbers are used in extracts from participants’ interviews. Each participant quote is presented with their number, gender (F for female, M for male), their group (OA for older adults, HCP for healthcare providers), and age (for the older adult group only).

**Table 3.** Themes.

**Theme 1**	Insufficient follow-up from healthcare providers left older adults to ‘fend’ for themselves
**Theme 2**	Providers should consider patients’ emotional and psychological health following a cardiac event
**Theme 3**	Support from family members facilitated rehabilitation
**Theme 4**	Medical and rehabilitation information should be tailored to patients’ needs
**Theme 5**	Spaces for participation in decision-making and more one-to-one sessions with healthcare providers are critical for older adults
**Theme 6**	A challenging healthcare journey during COVID-19

### Theme 1: Insufficient Follow-up from Healthcare Providers Left Older Adults to ‘Fend’ for Themselves

Older adult participants reported that they rarely were seen in person. They stated that the use of phone and video calls was the norm. Some older adult participants perceived comprehensive follow-up and communication with providers as very positive, in particular at the start of the rehabilitation process. However, some older adults reported ‘feeling alone’ or ‘on their own’, especially during the first two months after discharge.

“*I felt sick all the time, which was awful, and I called the surgeon, but I didn’t really get any help there […] I think, I think that first two months with no help, I felt quite alone there […] I just needed more contact the first two months”* (P17F/OA;77y.o.)

Most older adults expressed the need to receive accurate, timely and comprehensive discharge instructions, as well as information on how to engage in activities of daily living.

### Theme 2: Providers Should Consider Patients’ Emotional and Psychological Health Following a Cardiac Event

Having a contact person or someone to talk to was reported as helpful, often contributing to feelings of ‘being supported’ by healthcare providers. Support provided by nurses was especially highlighted by older adult participants as positively impacting their experiences.

“*She* [the nurse] *helps me, if I have any questions… I call her, and she is good. She is helping me in my exercise stuff and that's why I see her too.”* (P15M/OA;67y.o.)

Several older adults reported that a few healthcare providers’ attitudes were suboptimal. In particular, regarding their emotional state or their mental health. Feeling valued and supported was perceived as particularly important shortly after hospital discharge.

“*Because you’re not only a doctor to… prescribe pills… but you are also a doctor of emotional welfare, especially in this time* [pandemic]*. Well, he could have called me and say how are you doing* [participant's name]*? What can we do for you? No. nothing”* (P01M/OA;75y.o.)

During a member checking session, participant P01 M reported that there was a gap in terms of the mental health care he received, and what he called ‘body trauma’. As he expressed, ‘not wanting to live anymore’ was a concern that should have been addressed rapidly by a healthcare provider. Regarding mental health care and screening, a provider explained the challenges related to screening patients for mental health conditions.

“*So, sometimes there are clear symptoms, like flashbacks, irritability and aggression, and sleep disturbance and hypervigilance and hyperarousal. Those are the classic symptoms of PTSD […] but sometimes, is my impression that people may be sort of numbed out, they may be a little disassociated, flatten out emotionally speaking. So, when they come through intake screening in cardiac rehab, they might not score very highly at all, on the screening test, they might sort of fly under the radar”* (P21M/HCP)

Some older adult participants also reported feeling ignored by providers or feeling dismissed, these feelings were often associated with interactions with providers before and during hospitalization, and shortly after discharge.

### Theme 3: Support from Family Members Facilitated Rehabilitation

Family members offered invaluable support to motivate the participant to exercise and to engage in other cardiac rehabilitation activities. Their input during appointments was reported as very helpful by providers because they often remembered important information about their relative's care and provided great support when using technology and navigating the logistics of the appointments. In addition, family members helped providers find meaningful connections between rehabilitation activities and activities older adults enjoyed.

“*I think it's a huge support because they are there to support them, and even sometimes being on like, zoom, you know, we have like an older lady and she doesn’t know how to use it and her daughter joined with her to be able to support. So, yes, I think it's a huge support for them”* (P23F/HCP)

Some older adults reported, even though COVID-19 restrictions prevented them from being in the hospital with their loved ones, their family members would provide support in a variety of ways; for instance, by taking them to the entrance of the hospital and watch them from outside.

### Theme 4: Medical and Rehabilitation Information Should Be Tailored to Patients’ Needs

Older adults reported that understanding instructions regarding their medical care was very important, including both written and verbal information, in particular when information was easy to understand and when it addressed their concerns, such as self-care and how to take medications.

“*They* [providers] *sent me home with all the pills and when I had questions, I just asked, you know, why am I taking this pill? […] They did send me home with a book, as to what I should do when I’m home, and how I should care for myself”* (P12F/OA; 72y.o)

However, some older adult participants reported that information was insufficient, not tailored to specific needs, or difficult and overwhelming.

“*They* [providers] *have about three different things that they want me to complete. One for my exercise program, and the next one is my diet. And to me, all this information is just too much*” (P03F/OA;81y.o)

Barriers to obtaining relevant and sufficient information about medical care after surgical and non-surgical procedures were reported by older adults. In addition, not receiving tailored information and attention to their individual conditions, such as arthritis and anemia, in a timely manner was also reported as a concern.

### Theme 5: Spaces for Participation in Decision-Making and More one-to-one Sessions with Healthcare Providers are Critical for Older Adults

Several older adult participants reported engaging in shared decision-making with their healthcare providers was the preferred method to make decisions about their medical care and rehabilitation. According to providers, learning about the patient and their unique goals is an important first step to promote participation, particularly collaborative goal setting.

“*A big emphasis is put on goal setting, teaching participants how to set goals […] I help them set a goal so that they participate in the decision-making”* (P22F/HCP)

Some older adults reported barriers to participation in the decision-making process, as well as the perception that their care was ‘rushed’. Lack of spaces for participation was also reported. For instance, patient-centered one-on-one sessions with providers where older adults are free to express their concerns.

“*No, they* [providers] *never asked me, no, what my concerns were”* (P03F/OA;81y.o.)

Healthcare providers perceived that those in the oldest cohort had more difficulties related to meaningful involvement in decision-making; however, spaces for participation and expressing concerns are missing for some patients, reflecting an immense gap between the provision of care and what older adults perceive.

### Theme 6: A Challenging Healthcare Journey During COVID-19

Hospitalizations for older adult participants were impacted heavily by the pandemic and some older adults reported feelings of isolation and fear related to COVID-19, especially those who experienced outbreaks while in the hospital and could not have a family member with them.

“*I really needed my family and I got cut off from my family just because I got sick* [Covid-19]*, cause I ended up in the hospital for 33 days and then I went in for another 12 days more […] When I was in isolation my husband was not allowed in the hospital”* (P20F/OA;74y.o)

Delayed medical procedures, long waits to receive care and insufficient access to healthcare providers were reported as negative experiences by older adults. Older adults were also affected by public health measures such as distancing and the closure of recreation spaces. Lack of social interaction and adequate spaces to exercise contributed to feelings of isolation among older adults and hindered their active engagement in rehabilitation activities.

*It felt very lonely the first two months. Because of the pandemic, I couldn’t access what normally would be there*” (P17F/OA;77y.o)

Difficulties identifying providers while in hospital because of the use of protective equipment were also reported as stressful, as well as the constant fear of getting sick, as they witnessed other patients dying in their hospital unit.

## Discussion

Communication and care coordination are essential for the effectiveness of transitional care for older adults.^14^ Participants in this study reported that timely follow-up shortly after discharge and ongoing communication from healthcare providers contributed to a smooth transition from hospital to cardiac rehabilitation. This finding is consistent with previous literature on transitional care. As suggested by Naylor and colleagues,^15^ the healthcare provider who is the consistent point person in communication with older adults and their families has a critical role to ensure communication and trust during transitional care. In addition, healthcare providers can act as a ‘liaison’ to facilitate referral while providing information about the benefits of cardiac rehabilitation to individuals at the bedside.^16^

In the present study, in line with previous literature,^17,18^ a good and trusting relationship with providers, particularly nurses, facilitated a smooth transition from hospital to cardiac rehabilitation for older adults. Stolee and colleagues^19^ found themes related to trust and respect; family members who interacted with a healthcare provider who inspired trust were more likely to ask questions, take advice and be involved in the decision-making. However, for some older adults, interactions with providers were not viewed as positive, and negative experiences were often related to difficulties identifying and trusting providers.

Families were found to be a major facilitator for the participation of older adults in their care, particularly by encouraging them to adhere to lifestyle changes. Consistent with the literature, this finding highlights the relevance of family involvement in the transitional care of older adults, including participation in medical visits and decision-making, as well as helping older adults navigate multiple healthcare settings and medical appointments with providers.^20,21^

Older adults perceived a significant gap in the provision of psychological services and in some cases, lack of acknowledgement from healthcare providers regarding their mental health. For older adults living with cardiovascular disease, the prevalence of mental health conditions, particularly depression and anxiety,^22^ is higher compared to other age groups.^3^ Sever and colleagues^23^ noted that the characteristics of patients who have a new onset of depressive symptoms can influence the depression outcomes after cardiac rehabilitation. Given that the COVID-19 pandemic has exacerbated feelings of vulnerability, anxiety and helplessness for people living with cardiovascular disease,^24^ mental health supports for older adults are critical.

Engaging patients and their families in their care is an essential component of effective and comprehensive transitional care.^25^ However, sometimes opportunities for the active participation of older adults, are missing. There is a need for responsiveness in the care of older adults living with cardiovascular disease. Healthcare providers’ responsiveness when interacting with patients is critical, specifically by respecting autonomy, being flexible and adapting to the needs and expectations of their patients.^26^

The COVID-19 pandemic was reported as a major barrier by older adults, particularly after discharge. The pandemic contributed to gaps in communication, social isolation and delayed medical procedures. This finding aligns with current literature on COVID-19 related disruptions to acute care,^27^ including challenges such as poor interprofessional communication, information and communication breakdowns, insufficient documentation and updates in terms of discharge plans and low levels of patient and family engagement.

### Limitations

Key methods such as field immersion and observations were not used due to pandemic restrictions. The fact that data were collected remotely could have impacted the recruitment of participants who did not have access to digital devices, or those who are more comfortable establishing trust and rapport face-to-face. More male participants agreed to have an interview in the older adult group, and individuals over 85 year refused participation. Results from this study could have been different with a more diverse group of older adults (eg, cultural background, diverse gender identification, low income, etc.).

## Conclusion

This research study aimed to explore the perspectives of older adults and healthcare providers on transitional care from hospital to cardiac rehabilitation, with a special focus on patient in decision-making. While many participants reported very positive experiences, the findings of this study indicated a number of potential gaps in the provision of transitional care services as reported by older adults who had a cardiovascular event. Most of the potential gaps in care were reported to occur in the first few weeks post hospital discharge, a time that older adults described as vulnerable and lonely, with little to no support from healthcare providers.

Healthcare providers should be encouraged to pay attention to the emotional needs of older adults after hospital discharge. Applying the principles of liaised referral to cardiac rehabilitation for all^16^ and inclusive patient engagement^28^ can be a fruitful strategy to provide more comprehensive transitional care for older adults and ensuring that older adults have adequate spaces and opportunities for meaningful participation in decision-making; for instance, focusing on relationship building for older adults and their healthcare providers, through the development of trust, self-awareness, acceptance, understanding, education and communication.

This study highlighted the need for personalized care for older adults and the critical aspect of emotional support, attention to mental health and relationship building with providers during the transitional care process. In addition, offering more spaces and opportunities for the active participation of older adults in their care can contribute to the delivery of comprehensive, patient and family-centered care for older adults who live with cardiovascular disease.
